# High-gradient magnetic fields and starch metabolism: results from a space experiment

**DOI:** 10.1038/s41598-022-22691-2

**Published:** 2022-10-29

**Authors:** K. H. Hasenstein, M. R. Park, S. P. John, C. Ajala

**Affiliations:** 1 Biology Department, University of Louisiana at Lafayette, Lafayette, LA 70504-43602 USA; 2 Central Area Crop Breeding Div., National Institute of Crop Science, Suwon, 16429 Gyeonggi-do Korea; 3 Present Address: Cemvita Factory, 9350 Kirby Drive, Suite 100, Houston, TX 77054 USA

**Keywords:** Biological techniques, Biophysics, Plant sciences

## Abstract

Directing plant growth in weightlessness requires understanding the processes that establish plant orientation and how to manipulate them. Both gravi- and phototropism determine directional growth and previous experiments showed that high gradient magnetic fields (HGMF) can induce curvature in roots and shoots. Experiments with *Brassica rapa* verified that that gravitropism-like induction of curvature is possible in space and that the HGMF-responsive organelles are amyloplasts. We assessed the effect of space and HGMF based on 16 genes and compared their transcription with static growth and clinorotation. Amyloplasts size in root tips increased under weightlessness but decreased under clinorotation but not in response to magnetic fields. Amyloplast size changes were correlated with reduced amylase transcription in space samples and enhanced transcription after clinorotation. Mechanostimulation and weightlessness have opposite effects on the size of amyloplasts. The data show that plants perceive weightlessness, and that their metabolism adjusts to microgravity and mechanostimulation. Thus, clinorotation as surrogate for space research may lead to incorrect interpretations.

## Introduction

The perception of the gravity stimulus and the involvement of amyloplasts have long been the focus of plant biology because of the cryptic nature of essential steps in the response of a biological system to physical stimuli such as gravity^1–4^. While the first step of the gravitropic response primarily depends on the interaction of intracellular particles with Earth’s gravitational field, the ability to respond to the gravity signal depends on biological conditions such as auxin sensitivity and transport^5^, time^6,7^, the cytoskeleton^8^ and physical parameters such as temperature^9^, orientation^10,11^, osmotic conditions^12^, mechanical noise^13^, cytoplasmic viscosity^14^, and high-gradient magnetic fields^15–17^. The redundancy contributes to the stability of the graviresponse system and can integrate additional signals such as hydrotropism^18,19^ and touch^20,21^. Because any physiological response is a function of the accumulated signal (auxin, altered gene expression, pH, ion, or charge shifts), control of the response shifts from the original signal to downstream events such as signal processing (translation) and response variables that are related to signal strength and persistence^22^.

Therefore, the analysis of mechano-sensing and (gravi)response depends on the elimination of the gravity effects by experimenting under weightlessness conditions in space. The study of HGMF on amyloplast movements was attempted in ground studies^15,23–26^ and was the focus of a shuttle experiment in 2003 (STS-107)^27^. However, the disintegration of the Shuttle during reentry made the intended analyses impossible. Nonetheless, this experiment provided strong evidence that mechano-sensitivity increased in microgravity^27^.

Here we report data from a space experiment on the Space-X Crew Resupply Service 3 mission. The aim of this investigation was to repeat the ill-fated experiment on STS-107. However, instead of flax (*Linum usitatissimum*) we used *Brassica rapa* seeds to study the effect of magnetic gradients and to take advantage of its Arabidopsis-related genome to assess transcriptional responses of genes related to growth, metabolism, auxin, and stress. Because of the dual effort to characterize the effect of HGMF and transcriptional analysis the remainder of the introduction covers separately each aspect.

### HGMF

Induction of curvature by magneto-mechanical forces depends on the magnetic susceptibility (ϰ), which is characteristic of a given substance and describes its ability to acquire magnetization I as a result of the (inducing) magnetic field H such that I = ϰH. Diamagnetic substances have negative susceptibility and include the vast majority of biological substances. However, some metal-containing proteins such as hemoglobin, cytochrome, ferritin etc., can have positive susceptibility and are paramagnetic^26,28,29^. The magnetic susceptibility of dia- and paramagnetic compounds is proportional to their density and their behavior in a magnetic gradient is analogous to that in the gravity field^16,24,26^. Magnetic susceptibility and density of the cytoplasm are equal to that of water, ϰ _w_ = 7.2 × 10^–7^ emu (electromagnetic units, table value), and that of starch ϰ_st_ = (8 ± 0.2) × 10^–7^, thus the differential susceptibility between starch and cytoplasm Δϰ ≈ 8 × 10^–8^ emu. The density of starch ρ_st_ = 1.5 g/cm^3^, and cytoplasm ~ 1 g/cm^3^; therefore Δρ = 0.5 g/cm^3^ and Δp/Δ ϰ ≈ − 6 × 10^6^ (g/cm^3^)/emu^26^.

**Figure 1:**
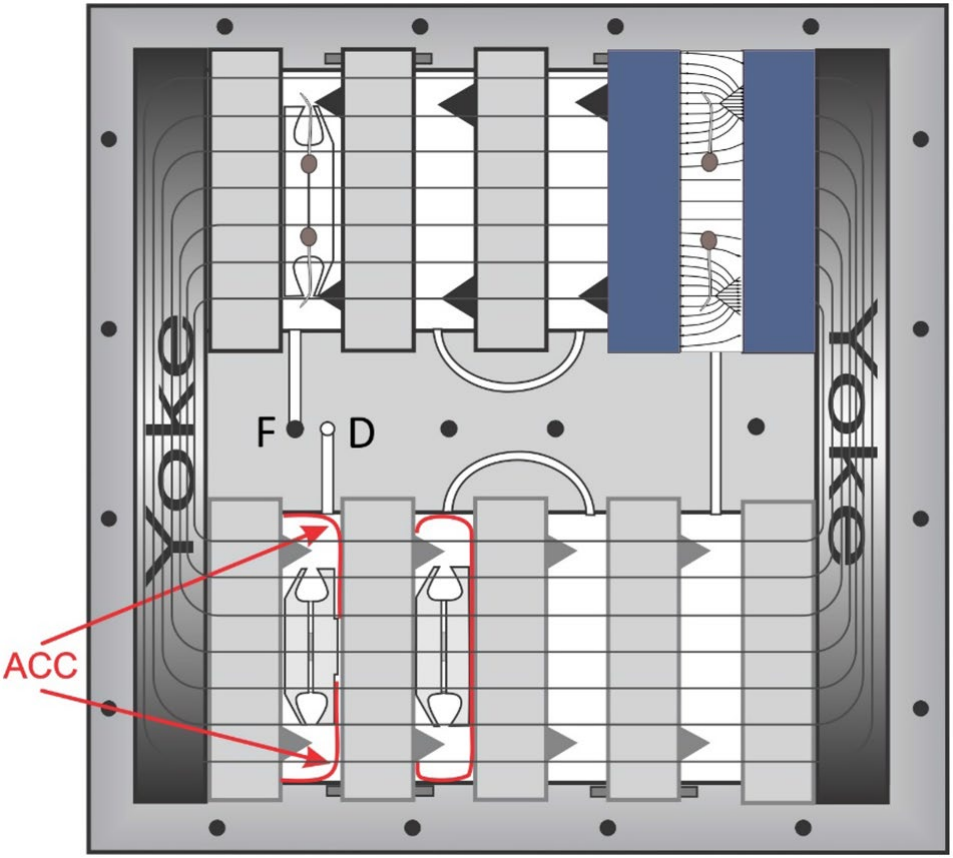
The design of the magnetic field chamber (MFC) with 10 magnets arranged in two stories with a total of eight compartments. The magnetic field was contained with two ferromagnetic yokes. Each compartment contained two ferromagnetic wedges and two pieces of activated charcoal cloth (ACC), and one seed cassette each. The diagram on the upper right shows the distortion of the magnetic field by the wedges (applicable to all compartments) and the projected growth of the seedling roots. The flow path of the fixative from a single fill port (F) to the drain port (D) is shown as white canals for front-visible paths; connections on the rear side between the individual compartments are shown as dark lines.

Although other methods of generating HGMF are available^24,26^, the generation of a magnetic gradient in the experiments described here, was realized by inserting into a uniform, strong magnetic field ferromagnetic wedges. The wedges become magnetized and create a HGMF (Fig. 1) such that the gradient ∇H is directed toward the tip of the wedge. Therefore, the force acting on a diamagnetic body (ϰ < 0) repels starch-filled amyloplasts from the inserted object. In contrast, a para- or ferromagnetic body (ϰ > 0) would experience an attractive force. The force generated by the HGMF utilized in the employed setup has been determined previously to be about 0.6 g^30^ and exceeds the acceleration that plants respond to (about 10^–3^ g^31^).

In contrast to cultivation on earth where orienting factors are provided by gravity as constant and light as intermittent influence, plant growth in space cannot rely on gravitational clues; it depends on the plants’ (intermittent) phototropic response. However, in the absence of light, especially root elongation is variable, and orientation responds mostly to water gradients (hydrotropism)^19,32^. Removing the two most consequential vectors for plant orientation, i.e., growing plants under weightlessness conditions in the dark, provides an opportunity to examine the role of amyloplasts for directional growth analogous to their role in gravity sensing. Probing magnetophoretic effects and the space conditions on root growth was the objective for the experiment described here.

The investigation included transcription analyses of 16 genes that represent auxin effects (*IAA5, PIN 1, 3, 7*) as the main growth regulator of differential elongation^33–35^, starch katabolism (*Amy1*)^36^, sucrose synthase (*SUS,* cell wall formation)^37^, *UBQ1* (ubiquitin1; a putative reference^38^ and indicator of protein metabolism)^39^, *TUB1* (tubulin1; reference^38^, cell growth, mitosis), *ACT7* (actin7; putative reference^40^, root growth and germination^41^), *ADH1* (alcohol dehydrogenase; stress response^42^), *COX* (cytochrome oxidase subunit1; metabolism, stress^43^), *G6PDH5* (glucose-6-phosphate dehydrogenase5; oxidative pentose phosphate pathway, basic metabolism^44^), *GLK* (glucokinase; carbohydrate metabolism^45^) HXK (hexokinase 1; glucose metabolism and developmental regulator^46^), *PFK* (phosphofructokinase; carbohydrate metabolism, hypoxia stress^47^), and *TAGL* (triacylglycerol lipase; lipid metabolism^48,49^). The transcription pattern of these genes under conditions such as space (weightlessness), clinorotation (enhanced mechanostimulation), and static controls provided information on how seedlings respond to the respective conditions.

This report describes an experiment that built on prior experience (i.e., Biotube1 on STS-107) but used genetically better-characterized plant material, *Brassica rapa*. Our data support HGMFs as suitable method to establish plant orientation in space but also show that mechanostimulation affects gravisensing-related metabolism. The report demonstrates that plants perceive weightlessness (“micro-gravity”) and respond by enhanced starch production in gravity perceiving cells.

## Material and methods

### Plant material

*Brassica rapa, var. rapa* seeds were commercially sourced. No approvals were required for the study, which complied with all relevant regulations. Seeds were attached to germination paper with polyvinyl acetate (clear Elmer’s) glue such that the micropyle was oriented toward the opening of seed cassettes (Sup. Fig. 1). The seeds cassettes were designed to fit into specially designed magnetic field chambers (MFCs, Fig. 1).

### Experimental setup

The experiment was a follow-up of the STS-107 experiment that was lost as a result of the shuttle accident in 2003. A computer (PC104 stack) controlled all aspects and was remotely operated such that no crew involvement was necessary. The experimental principle of the hardware (Fig. 1) and the arrangement of the components is shown in Sup. Fig. 2. Experiment initialization, water dispensation, image acquisition, temperature and pressure recording, and termination of the experiment by fixation were remotely initiated and controlled by the hardware’s own computer. The entire hardware was custom built at the Kennedy Space Center using several iterations and named Biotube-MICRo (**M**agnetic **I**nduction of **C**urvature in **Ro**ots).

### Hardware

The MFCs consisted of machined aluminum cases each containing 10 Neodymium Iron Boron (NdFeB) magnets, 12.7 mm thick with a magnetization of about 30,000 Oe (~ 3 Tesla, Magnet Sales and Manufacturing, Culver City, CA). The magnetic circuit was closed by two yokes. To the surface of each magnet, two wedges of ferromagnetic steel (equilateral cross section, 6 mm high, 50 mm long) were attached that generated the magnetic gradients (Fig. 1). Between each pair of magnets seed cassettes were inserted that contained 10 *Brassica rapa* seeds. Five seeds each were glued (Elmer’s clear glue) along the long edges of germination paper. Roots that emerged from the seed cassettes were expected to curve away from the wedges.

A camera system consisting of eight cameras on either side was installed between two of the three MFCs such that the cameras were positioned opposite a window that permitted viewing the seed cassettes. Illumination was achieved by a single IR LED (750–800 nm) to avoid phototropic stimulation. The LEDs were activated only during imaging. The cameras acquired images in two-minute intervals and the video signal together with temperature, and atmospheric pressure data and position number were transmitted to the Kennedy Space Center (Florida) as well as recorded internally on a hard drive.

### Space flight parameters

The experiment was launched on Space-X3 (April 18, 2014) and initiated after 19 days by dispensing 400 μL deionized water to each seed cassette from a Micro Effusion Device for Space Applications (MEDUSA). The seeds germinated after about 22 h. The experiment was terminated after 48 h by the injection of RNA-Later® into MFC-C (first in the fixation sequence, not imaged), injection of 4% formaldehyde in PHEMD buffer^50^ in MFC-A (image sequences designated ‘Y’). MFC-B received RNALater, was designated ‘Z’ and third in the fixation sequence. MFC-C did not contain magnets but Al blocks of identical dimensions as the magnets and served as control. The dispensation of the respective fixative was initiated by pressurizing an aluminum chamber that contained sealed bags with fixative, one for each MFC. A solenoid valve controlled fixative flow to one MFC at a time. Excess liquid was collected in a second bag housed in a plastic cage. The entire hardware and fixed plant material deorbited on May 20, 2014. After 2 days the hardware was received at the Kennedy Space Center, and sample processing was completed during the next 3 days. A ground control using the space hardware was performed at the Kennedy Space Center on a large clinostat (8/12–8/15/14, 1.5 rpm) and at the University of Louisiana using single clinorotated MFCs without imaging but under otherwise identical conditions.

### Data analysis

Image sequences were obtained from HD-stored files and compiled as video sequences (Sup. Video 1 and Sup. Video 2). Root curvature was recorded from seed cassettes that were submersed in the same buffer as during fixation in an upright position and photographed from four sides. After disassembly of the seed cassettes, root appearance was categorized as germinated, straight, or curved. Curvature was evaluated as affected by HGMF only if the root length was sufficient to reach the HGMF area and corresponding curvature was visible. Only roots that were exposed to HGMF were used for the transcriptome analysis.

Microscopy was performed on seedlings fixed in 4% (v/v) formaldehyde in PHEMD buffer^50^. Samples were dehydrated in a graded ethanol series and 100% acetone and embedded in Spurr’s resin. Longitudinal median sections (2 μm thick) were cut on an ultra-microtome (Sorvall MT2-B) and stained with toluidine blue (0.1% [w/v] in 0.1% [w/v] boric acid). The serial sections were photographed with a digital camera (Sony DKC-ST5) and measured using ImageJ (v.1.53).

RNA extraction was performed in four separate sections of seedlings (root tip, root proper, hypocotyl, and cotyledons) using a kit (Spectrum™ Plant Total RNA Kit, Sigma STRN250) following the manufacturer’s protocol. Transcription analysis was performed after reverse transcription (High-Capacity cDNA Reverse Transcription Kit, Applied Biosystems, USA) and qPCR reactions for 16 different genes using specific primers, with stable and suitable efficiencies (Sup. Table 1). Conditions for the amplification on a Step-one real-time PCR system (ThermoFisher Scientific) included a 2 min incubation at 95 °C, followed by 40 cycles (95 °C for 10 s; 60 °C for 10 s) with fluorescent readings taken at the end of the annealing cycle. Quality control included melt curve analyses and capillary electrophoresis (QIAxcel Advanced).

The assessment of the space environment on seedling biology was examined using qPCR data of 16 genes. Typically, qPCR data rely on so-called reference or housekeeping genes, but this approach does not consider that the environmental conditions (space flight, clinorotation, and static growth conditions) are likely to affect reference genes themselves. Therefore, we compared the entire data sets against each other based on all studied conditions. If no difference exists between the two data sets, then such plots result in a diagonal line with a slope of one. The evaluation based on slope and variance (R^2^ value) as indicator of overall stability of gene activity also provides information on significantly affected genes. Such data points will show up as ‘outliers’ from the bulk distribution^51^. This approach was evaluated based on four individual tissues of the seedlings, resulting in 64 data points (16 genes × 4 tissue types), for each treatment.

The most relevant comparison of the explained type focuses on the effect of weightlessness and HGMFs in addition to the effect of clinorotation and static, 1 g controls. The complete set of comparisons is provided as Sup. Table 2. Transcription data were evaluated based on correspondence plots of pairwise comparisons. Significant changes in transcriptions were evaluated by the Mean Method^51^, and statistical significance was based on Z-scores.

## Results and discussion

### Growth and seedling development

Each MFC contained eight seed cassettes with ten seeds each. Seed germination in the two MFCs with HGMFs was higher than in the non-magnetic chamber (Table 1), indicating that the presence of magnets and magnetic fields did not negatively affect germination.

**Table 1:** Germination and root length of *Brassica rapa* seeds in magnetic field chambers with (A&B) and without magnets (C).

			Cassette:							
			1	2	3	4	5	6	7	8
MFC-A (HGMF)	Average:	14.3	14.6	15.4	11.4	14.4	15.1	16.2	12.6	14.7
	SE:	4.2	3.2	3.5	3.7	4.4	2.8	4.8	4.8	5.0
	N =	77	9	10	10	10	10	10	9	9
MFC-B (HGMF)	Average:	16.1	16.6	15.0	17.3	16.8	15.5	16.3	16.4	14.6
	SE:	3.5	2.5	3.0	3.6	4.9	3.5	3.7	4.0	2.8
	N =	80	10	10	10	10	10	10	10	10
MFC-C (no magnets)	Average:	14.7	19.1	14.2	13.4	15.2	15.3	14.5	13.0	13.4
	SE:	4.4	3.9	4.7	4.0	2.3	4.3	6.4	3.3	3.2
	N =	70	8	10	9	8	9	10	7	9

There was no difference between chambers or seed cassettes.

### Curvature induction by HGMF

**Figure 2:**
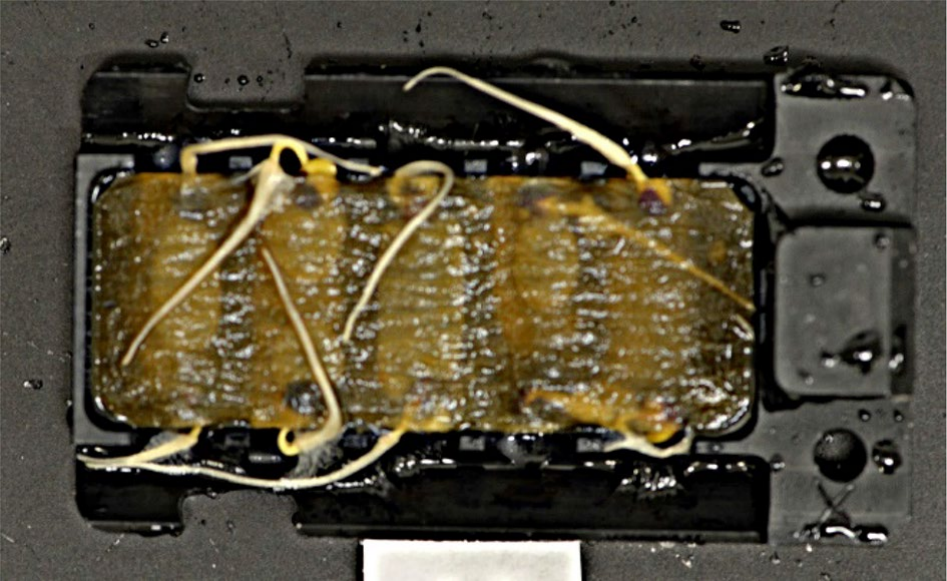
Example of root orientation inside seed cassettes. The predominant curvature and elongation occurred onto and along the surface of the germination paper.

Exposure to HGMF induced curvature (Sup. Video 1, Table 2). However, substantial curvature occurred in both presence and absence of magnetic gradients. The non-magnetic chamber showed 81% curvature in roots within the seed cassette around the germination paper (Fig. 2).

**Table 2:** Germination and curvature in the presence and absence of HGMFs of a total of 80 seeds per MFC.

	Not germinated	Straight^a^	Curved	Curved (HGMF)
MFC-A (HGMF)	3	8	40	28
MFC-B (HGMF)	0	0	50	30
MFC-C (No magnets)	10	5	65	0

^a^Refers to roots that never emerged from the seed cassette or were too short to reach the HGMF area (as shown in Fig. 2).

MFCs with magnets showed curvature that was independent of the HGMF because of growth patterns or curvature occurring inside the seed cassettes where the magnetic gradient was too weak to affect curvature.

**Figure 3:**
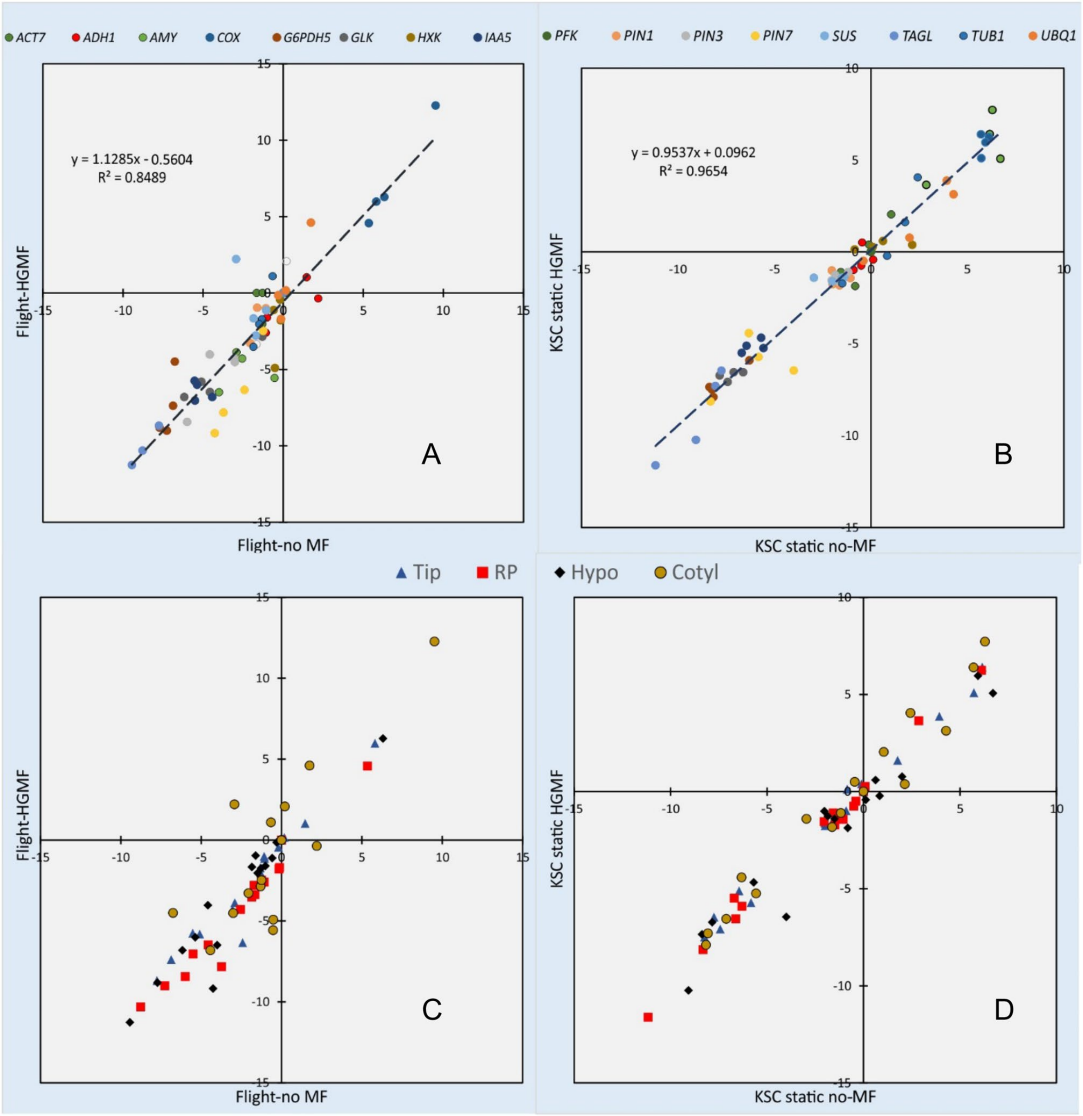
Transcription values between space grown *Brassica rapa* seedlings (**A**, #1) and static ground controls (**B**, #23) in the presence and absence of HGMFs show strong correlation (unity slope) and low scatter (R^2^ = 0.85 and 0.95), indicating no or low effect of magnetic fields on transcription. Panels (**C**) and (**D**) identify the examined tissues in (**A**) and (**B**), respectively. The data sets use *PFK* as reference and show efficiency-corrected ΔC_q_ and log(2) transformed values.

The comparison of space-grown or static ground control seedlings exposed to HGMF with seedlings grown in the non-magnetic field chamber (#1 and #23 in Sup. Table 2) shows strong correspondence between the two conditions (Fig. 3), indicating that magnetic fields do not affect general metabolism or transcription activity. This notion is in line with reports that failed to detect effects of magnetic fields on growth^52,53^ but contrasts with effects of weak magnetic fields on root curvature^54^ and effects of the geomagnetic field on stress response and hormesis^55,56^. The geomagnetic field (typically about 0.5 G) is orders of magnitude weaker than the employed magnetic fields in this research (ca. 30 kG).

These data indicate that factors other than HGMF induce curvature and the most likely factor is hydrotropism^18,32,57^. The lack of a gravity stimulus and the distance between the germination paper and the HGMF-generating wedges resulted in a large number of roots confined to the germination paper. The same factor also applied to seedlings in the magnetic chambers and resulted in reduced numbers of roots reaching the HGMF. However, the unambiguous evidence for curvature in space (see Sup. Video 1) and clinorotated seedlings (Sup. Video 2) indicates that HGMFs do have the ability to induce curvature as had been demonstrated earlier in roots, coleoptiles, inflorescences, and hypocotyls^16,17,26,58,59^.

The presumably hydrotropic growth reduced the number of roots that reached the influence of the HGMFs. Therefore, the overall number of roots curving in response to the magnetic gradient was low (Table 2).

**Figure 4:**
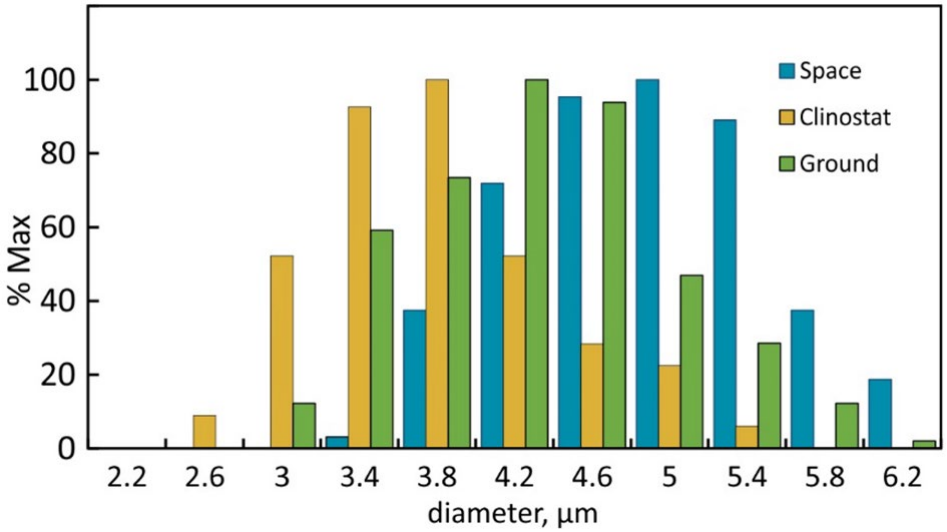
Size distribution of amyloplasts in the root cap of Brassica rapa seedlings grown in space, on the ground or in a clinostat. The data were compiled from 2 μm thick longitudinal sections of root cap tissue after fixation in paraformaldehyde. The difference between space and clinostat amyloplasts was highly significant (P < 10^–4^); the difference between static ground control and clinorotated amyloplasts was significant at the 5% level. Data were normalized based on 290, 243, and 210 amyloplast measurements for space, clinorotated, and static ground control plants, respectively.

Examining the gravisensing mechanism includes measuring the size of the presumptive sensors as previous work has shown that gravisensitivity depends on the amount of starch in amyloplasts^60^. Assuming equal density, the diameter of amyloplasts determines the relative mass. Measurements of amyloplasts in columella cells differed between the space-grown, clinorotated, and statically grown ground controls (Fig. 4) and indicates that the amyloplast size and therefore the gravisensing mechanism is responsive to the growth condition, notably weightlessness and clinorotation that respectively reduces and enhances mechanostimulation.

Earlier analyses of starch from space and ground controls found that ethylene reduces starch particle size and that in cotyledons starch particles sizes in space and ground controls were of equal size^61^. Since we used activated charcoal cloth to absorb volatile organic compounds (Fig. 1), we assume that ethylene was not a factor in the different experiments. In addition, we examined the size distribution of amyloplasts in the root columella. Earlier work showed that amyloplasts in gravisensing tissues (root cap and endodermis) are about twice the size of other tissues^7^. Our data (Fig. 4) indicate that the amyloplast size is responsive to the gravitational and mechanical stimulation. The reduced size after clinorotation, the average size in static 1-g controls, and the enlarged size in roots grown under ‘micro-gravity’ conditions support the notion that the extent of gravitational stimulation is inversely proportional to the mass of the amyloplasts. Thus, plants not only perceive the direction of an accelerative force but adjust their (starch) metabolism according to the amount of stimulation. If this concept is correct, then it should be supported by gene transcription data. The following analyses confirm that amylase activity indeed is affected by mechanostimulation.

### Transcript analyses

**Figure 5:**
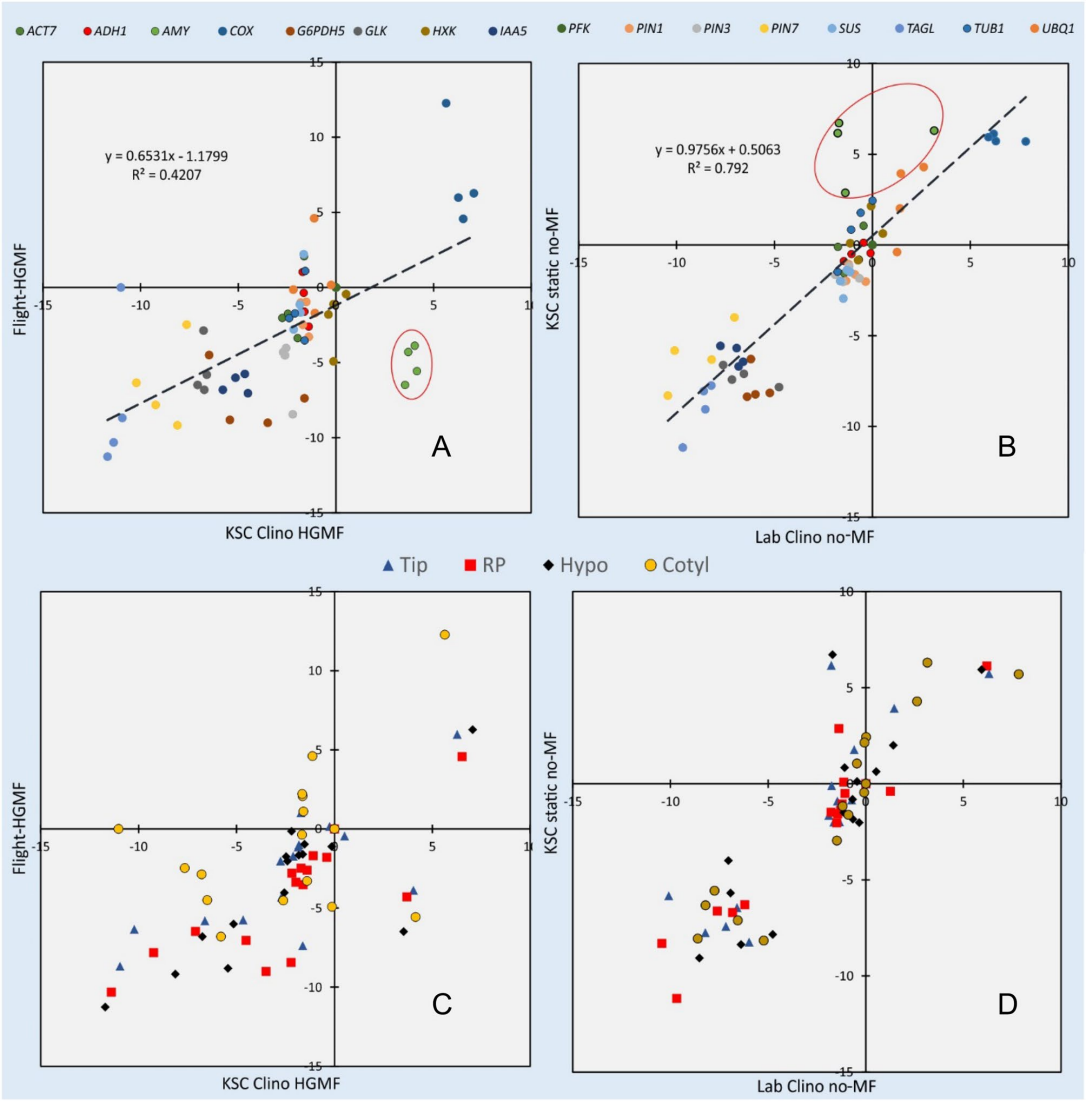
Transcription values for space grown and clinorotated KSC ground controls. *Brassica rapa* seedlings (**A**, #2) show strong reduction of *AMY1* transcription in clinorotated seedlings (circle). A comparison between static and clinorotated seedlings (**B**, #27) shows higher *AMY1* transcription in non-clinorotated seedlings. Panels (**C**) and (**D**) identify the tissues in (**A**) and (**B**), respectively. The circled data indicate *AMY1* transcription relative to *PFK*. In both cases average *AMY1* transcription was significantly different (p = 0.002, left and p < 0.001, right; n = 64). The data sets use *PFK* as reference and show efficiency-corrected ΔC_q_ and log(2) transformed values.

Since four different tissue types were analyzed, tissue variability and response to spaceflight and clinorotation can be assessed for all examined tissues and genes. Based on distributions of transcription patterns (Figs. 3 and 5), a comprehensive analysis of all treatment and tissue combinations was performed such that the scatter for each comparison and gene was determined based on the formula

$$\sum_{i=1}^{n}\sum_{j=1}^{4}\sqrt{{\left({X}_{ij}-\overline{{X }_{j}}\right)}^{2}+{\left({Y}_{ij}-\overline{{Y }_{j}}\right)}^{2}}$$

where j represents the tissue types (root tip, root proper, hypocotyl, cotyledons) and i the individual experimental comparisons (28, Sup. Table 2) or examined genes (16). This value was calculated for each analyzed gene and the smallest value (scatter in Figs. 3 and 5) was identified as the most stably transcribed gene (Table 3).

**Table 3:** Assessment of the consistency of transcription data based on the average of examined tissues.

	Gene	n	%
All tissues	*PFK*	60	13.4
	*PIN1*	47	10.5
	*TUB1*	45	10.0
	*GLK*	33	7.4
Root tip	*PIN1*	43	9.6
	*PIN3*	41	9.2
	*SUS*	40	8.9
	*GLK*	35	7.8
Root proper	*COX*	51	11.4
	*ACT7*	42	9.4
	*SUS*	42	9.4
	*HXK*	39	8.7
Hypocotyl	*HXK*	42	9.4
	*ACT7*	37	8.3
	*SUS*	37	8.3
	*ADH1*	27	6.0
Cotyledons	*PFK*	49	10.9
	*ACT7*	45	10.0
	*ADH1*	40	8.9
	*PIN7*	35	7.8

The top four choices of all combinations (as in Sup. Table 2) are based on the percentage that resulted in the least scatter of all 448 combinations (16 genes by 28 comparisons).

The distribution of the least variable gene transcription varied greatly (Table 3). Although common reference genes (e.g., *TUB1, ACT7*) are represented (Table 3), the observation that individual tissues differed in the genes of greatest stability and that the average of all tissues resulted in different assortments suggested that referring transcription data to the average of all measurements (i.e., the regression lines in Figs. 3 and 5) is superior to relying on a single gene. Because *PFK* showed the greatest stability for all tissues, we placed *PFK* at the origin of the coordinate system (0/0). However, the results explained below are independent of this selection.

The comparisons (Sup. Table 2) show the least and most significant effect on gene expression and can be used to identify the conditions that induce physiological responses in brassica seedlings. The greatest stability in gene transcription was seen for treatments with similar mechanical load, for example, static growth with and without HGMF, or clinorotated samples (KSC and Lab). The largest scatter or least consistent transcription pattern was associated with different mechanical loads such as flight (i.e., no mechanical load) and clinorotation (enhanced mechanical load), or static and space flight conditions. The main conclusion of these evaluations is that HGMFs or more generally, strong magnetic fields, do not affect transcription; HGMF data are equally present in the most and least affected conditions (Table 4). However, a comparison between clinorotated experiments with the original flight hardware at KSC and experiments in our lab (comparison # 17 and # 22) show statistically significant differences (Sup. Fig. 4).

**Table 4:** The similarity of gene transcription between pairs of treatments based on high gradient magnetic fields (HGMF) or no magnetic fields (no-MF) that were applied during the space flight (Flight), on ground controls (static), or during clinorotation (Clino) at the Kennedy Space Center (KSC) or in the laboratory (Lab).

	#^a^	R^2^, %	STDEV,%	Comparisons (all genes)
	23	94.8	1.9	KSC static no-MF *vs.* KSC static HGMF
	17	90.8	2.7	Lab Clino HGMF *vs.* KSC Clino HGMF
	28	82.6	7.5	Lab Clino no-MF *vs.* Lab Clino HGMF
	1	80.2	7.2	Flight-no MF *vs.* Flight-HGMF
	15	80.0	3.3	KSC static HGMF *vs.* KSC Clino HGMF
	18	78.8	3.7	Lab Clino no-MF *vs.* KSC Clino HGMF
	16	76.6	3.3	KSC static no-MF *vs.* KSC Clino HGMF
	25	76.3	2.6	Lab Clino no-MF *vs.* KSC static HGMF
	27	75.6	4.7	Lab Clino no-MF *vs.* KSC static no-MF
	24	73.1	5.2	Lab Clino HGMF *vs.* KSC static HGMF
	19	72.8	7.0	KSC static HGMF *vs.* KSC Clino no-MF
	26	70.7	5.5	Lab Clino HGMF *vs.* KSC static no-MF
	20	70.4	6.7	KSC static no-MF *vs.* KSC Clino no-MF
	14	60.6	7.7	KSC Clino no-MF *vs.* KSC Clino HGMF
	13	59.2	11.1	Lab Clino no-MF *vs.* Flight-no MF
	22	56.9	6.2	Lab Clino no-MF *vs.* KSC Clino no-MF
	21	56.2	7.3	Lab Clino HGMF *vs.* KSC Clino no-MF
	7	56.1	11.2	Lab Clino no-MF *vs.* Flight-HGMF
	12	53.6	7.0	Lab Clino HGMF *vs.* Flight-no MF
	9	52.3	10.0	KSC Clino no-MF *vs.* Flight-no MF
	10	51.0	8.2	KSC static HGMF *vs.* Flight-no MF
	11	50.7	8.4	KSC static no-MF *vs.* Flight-no MF
	6	49.9	7.5	Lab Clino HGMF *vs.* Flight-HGMF
	8	46.0	7.1	KSC Clino HGMF *vs.* Flight-no MF
	2	40.6	6.7	KSC Clino HGMF *vs.* Flight-HGMF
	5	38.0	7.4	KSC static no-MF *vs.* Flight-HGMF
	3	37.4	10.6	KSC Clino no-MF *vs.* Flight-HGMF
	4	37.4	10.6	KSC static HGMF *vs.* Flight-HGMF

The values were obtained by averaging the R^2^ values that resulted from using each of the 16 genes as reference (i.e., origin in graphical comparisons such as Figs. 3 and 5 and Sup. Figs. 3 and 4).

^a^Numbers refer to Sup. Table 2.

### Sensitivity of *AMY1*

Data sets comparing space-grown with clinorotated seedlings show large scatter and offset (Fig. 5A and Sup. Fig. 3A), suggesting that the shift in the transcription pattern is not dependent on HGMF but differences in mechanostimulation (i.e., clinorotation). This effect is especially noticeable for *AMY1*. The effect of clinorotation on amylase transcription is independent of the HGMF because transcription of *AMY1* in the absence of HGMF was higher in static seedlings than clinorotated seedlings (Fig. 5B). Space flight and clinorotation resulted in reduced and elevated transcription, respectively. A similar pattern was observed between clinorotated and static samples; however, HGMF had no effect (Sup. Fig. 3). The altered *AMY1* transcription between clinorotation at KSC and our lab is related to different stabilities of the hardware. The flight hardware contained a webbing-like base mount (implemented because of weight concerns), which provided stable support during space flight but flexed readily during clinorotation compared to a rigid assembly on the laboratory clinostat.

The transcription data correspond with the observed size distribution of amyloplasts (Fig. 4) and indicate that amyloplast size is regulated by starch degradation in clinorotated seedlings and amylase repression (starch accumulation) in space samples. Together these observations indicate that plant adapt to the weightlessness of space by increasing their amyloplast size which likely enhances their gravisensitivity. In contrast, clinorotation represents excessive mechanostimulation and leads to a reduction of amyloplast size through enhanced degradation (amylase transcription).

The observed *AMY1* levels between space-grown and clinorotated seedlings were independent of reference genes; the effect persisted regardless of whatever gene was used as reference. This observation supports using transcription of all available genes as a reliable approach to identifying transcriptional changes of individual genes.

The *AMY1* data correspond to earlier observations of enhanced gravisensitivity of space-grown lentil seedlings^62,63^ and reduced starch after clinorotation^64^. Although the STS-107 flight experiment could not be retrieved to measure amyloplast size, the image analysis of flax seedlings indicated that the magnetic gradient had stronger effects than during previous ground experiments because the root curvature started at a greater distance from the HGMF-inducing wedge^27^ than during ground controls. This observation is in line with the present data and strongly supports greater (gravi)sensitivity of plants growing in a microgravity environment.

The current report is the first to associate starch metabolism with amyloplasts size and gravisensitivity. The larger amyloplasts in space-grown plants suggest that the application of HGMFs in space is more effective than in earth-grown and especially in clinorotated plants. However, the unreliable growth direction of roots makes HGMF difficult to implement.

Gravisensitivity has also been linked to changes in calcium in statocytes^65–67^ and calcium has been shown to stabilize α-amylase^68–70^. Therefore, Ca^2+^ and amylase are controlling element for the starch content in statocytes. However, starch content is the result of homeostasis for catabolic and anabolic metabolism. Starch biosynthesis depends on a complex set of enzymes that include phosphoglucose isomerase (PGI)^71^, phosphoglucomutase (PGM)^72,73^, and starch synthases (SSs)^71,74^ among others. Data on the balance between starch degradation and starch biosynthesis undoubtedly would provide a more comprehensive assessment of the sensitivity of starch metabolism to reduced and enhanced mechanostimulation. However, the limited set of transcriptionally analyzed genes prevents a more thorough assessment of starch biosynthesis in response to altered mechanostimulation. Future space experiments might remedy this shortcoming.

### Genes other than *AMY1*

**Figure 6:**
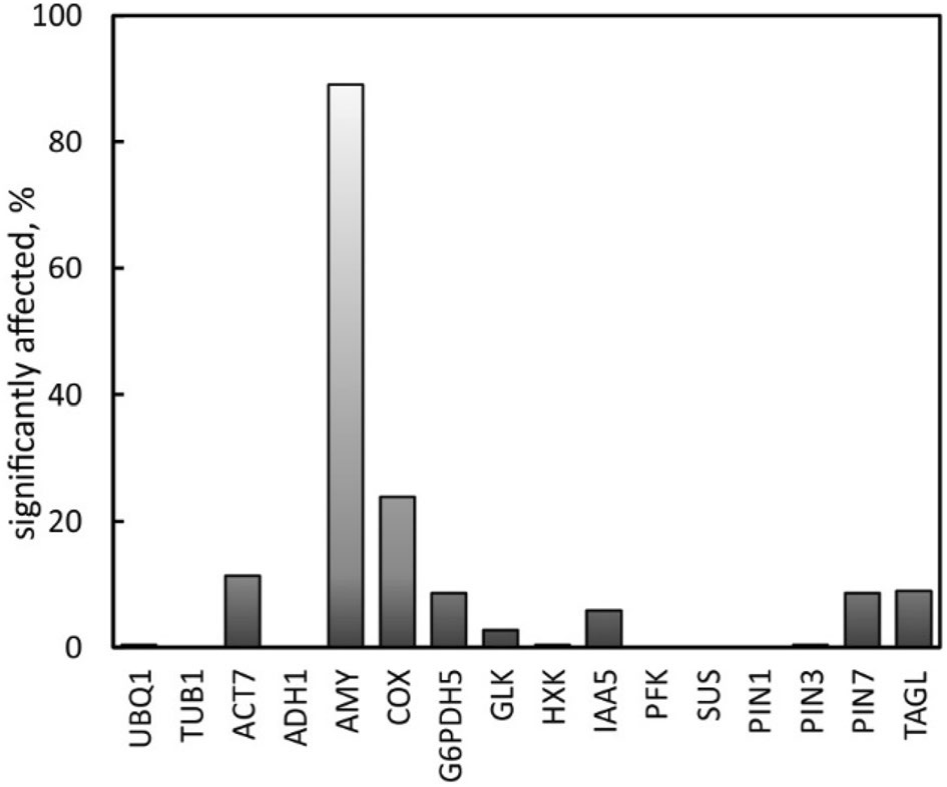
The percentage of significant (P < 0.05) changes in gene transcription in response to space, HGMF and clinorotation of *Brassica rapa* seedlings. Compared to *AMY1,* all other genes showed fewer significant alterations (% of 16 genes × 16 references).

Although weightlessness is the dominant difference between ground and space flights, the lack of density-driven gas exchange and water distribution are equally significant alterations for plant growth in space. Because the flight hardware was enclosed in a hermetically sealed (triple-contained) chamber, atmospheric effects can be excluded. Therefore, the following considerations only apply to gravity effects. Comparing the number of significantly (P < 0.05) affected combinations (genes by reference), shows the largest effect on *AMY1* transcription (Fig. 6).

Genes other than *AMY1* responded to specific conditions but at reduced power. *ACT7* showed modifications only when comparing clinorotation at KSC with static growth (Comparisons #19 & #20); *ACT7* was not affected by HGMF but sensitive to vibrations (higher frequency movements in excess to the 1.5 rpm of the clinostat). This relationship is similar to the enhanced AMY1 transcription addressed in Sup. Fig. 4. The enhanced oscillation can be verified by the jitter in Sup. Video 2. To improve visibility, the individual frames were aligned, but the inconsistent position of imprinted data confirms the added vibrational stimulation that affected *ACT7* activation (and enhanced *AMY1* transcription) compared to regular (smooth) clinorotation.

*COX* showed the strongest upregulation in space flight material compared with static growth conditions (#4, #5, #10 #11) but was not affected by HGMF. This observation further supports the claim that magnetic fields do not affect transcription. Instead, COX responds to space flight associated stress as has been shown previously for fish brain^75^ and skeletal muscle^76^. Changes in *G6PDH5* were limited to treatment differences between space flight and clinorotation (#8, #12, #15, #16) and correspond to earlier reports of altered enzyme activities of pine seedlings after exposure to clinorotation and hyper-g^77^ and enzyme activities in artemia cysts after space flight^78^. *IAA5* and *PIN7* responded to HGMF both under flight (*PIN7*) and clinorotation (*IAA5*) and underscores their relevance for auxin modification of growth. Although the number of roots that curved in response to HGMF was low (Table 2), it is possible that differential growth (curvature in response to hydrotropism?) affected the transcription of these genes. GLK did not respond significantly; changes were not limited to any particular condition and therefore cannot be associated with specific experimental conditions but likely represent natural variations. *UBq1, Tub1, ADH1, HXK, PFK, SUS, PIN1* showed no significant changes in transcription and therefore could all serve as references. The lack of response of *ADH1* is surprising as this gene was previously identified as space stress indicator^79,80^. TAGL is the only gene that shows reduced transcription in the presence of magnetic fields but only in clinorotated samples. While there is precedence that the lipid metabolism is affected by hypergravity^81^ and clinorotation^82^, this is the first observation that magnetic fields might contribute to such changes.

In summary, our data support plant proprioception of weightlessness and metabolic control of amyloplast size. The adjustment of size and mass of amyloplasts indicates that plants perceive gravity and ponderomotive forces, which provide not only enhanced gravisensitivity but also explain some metabolic responses to space conditions. The advantage of a sealed environment suggests that this effect is not an artefact but related to gravitational and mechanosensory responses. The results also demonstrate that HGMF do not influence gene transcription. Future work needs to investigate the role of other starch-related genes to understand the entire dynamic of metabolic plasticity that relates to weightlessness.

## Supplementary Information

Supplementary Video 1.

Supplementary Video 2.

Supplementary Information 1.

## Data Availability

All data are available upon request from the corresponding author.
