# Supplementary material for: High-gradient magnetic fields and starch metabolism: results from a space experiment
Source: Sci Rep. 2022 Oct 29;12:18256. doi: 10.1038/s41598-022-22691-2 (PMC9617909; doi:10.1038/s41598-022-22691-2)
Supplement: Supplementary file 3 — Supplementary Information 1. [file 41598_2022_22691_MOESM3_ESM.docx]

upplementary Information:


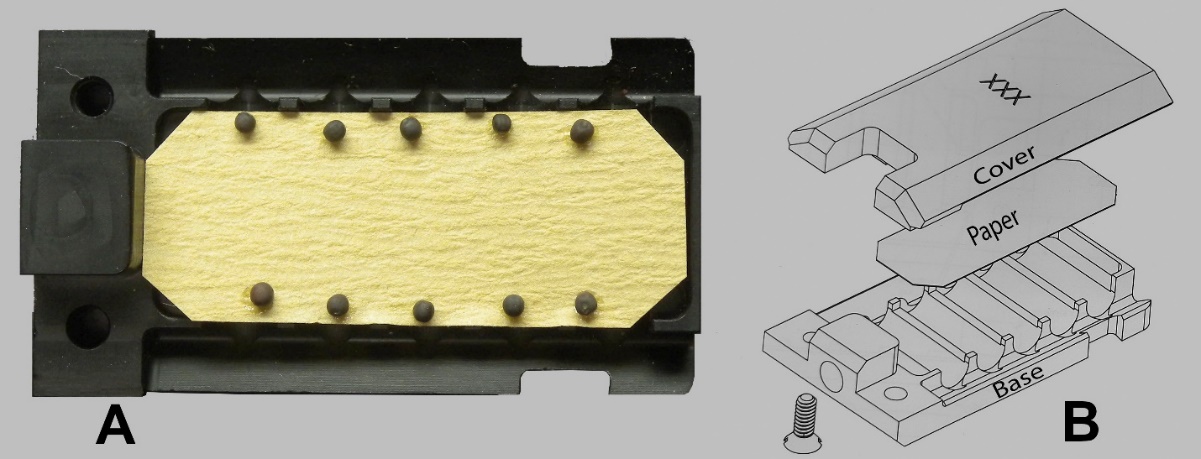
 **Supplementary Figure 1:** Arrangement of *Brassica rapa* seeds on germination paper inside a seed cassette (A) and a drawing of the complete cassette (B) with a nylon-screw secured cover (to not interfere with the magnetic field).


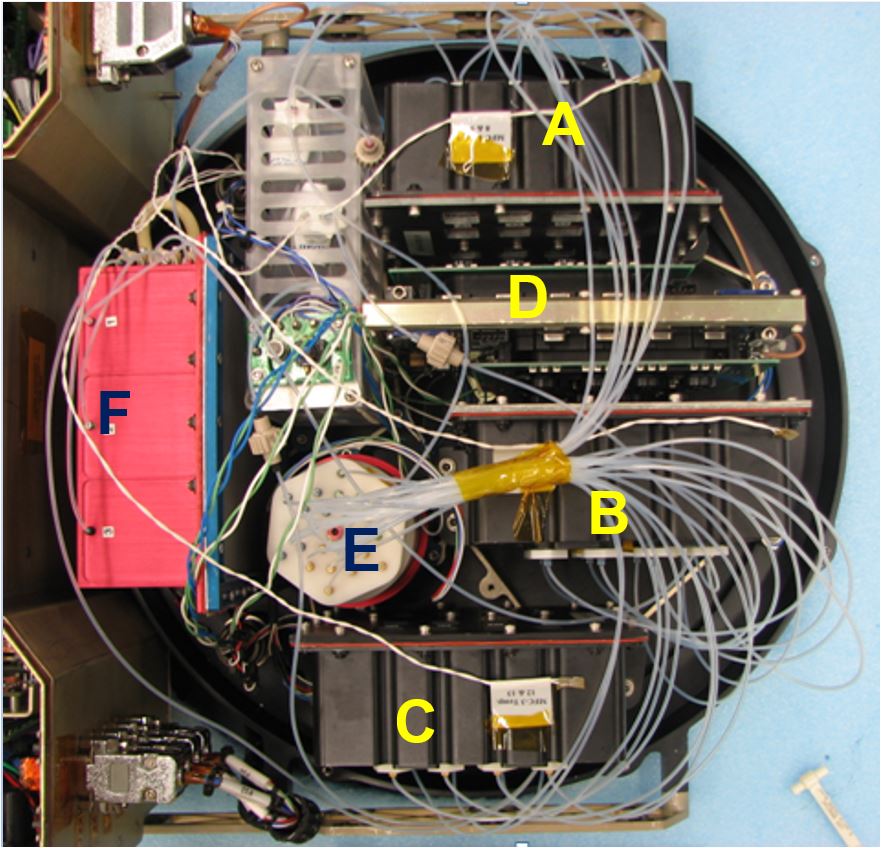
**Supplementary Figure 2**: The layout of the Biotube-MICRo experimental hardware (top view). Two chambers were equipped with 10 magnets each arranged in two stories (A & B). Chamber C contained non-magnetic aluminum blanks and served as control. The imaging system (D) contained 16 individual cameras with an IR-LED as light source. The experiment was initiated by dispensing water from the MEDUSA (E). Fixation resulted from pressurizing container (F) and sequentially activating solenoid valves to dispense fixative to chambers A, B and C.

**Supplementary Table 1:** Primer sequences (5’ to 3’) and efficiencies of genes used for qPCR analysis of RNALater fixed tissue samples.

| Gene | Accession | Forward | Reverse | Amp length | Efficiency |
| --- | --- | --- | --- | --- | --- |
| *ACT7*  *ADG*  *AMY*  *COX*  *GLK*  *G6PDH5*  *HXK*  *IAA5*  *PFK*  *PIN1*  *PIN3*  *PIN7*  *SUS*  *TAGL*  *TUB1*  *UBQ1* | NM_121018.3  AT5G48300.1  AT1G76130.1  NM 118632.3  AT 3G20040  AT3G27300.1  AF454961.1  NM_101427.4  AT1G12000.1  AJ132363.1  AJ249298.1  NM_179369.1  NM_122090.3  AT5G04040.1  D78496.1  Z24738.1 | AGCTTCGTGTTGCACCTGAA  GGAGCTAATGACTTTGGAAGTGAAGT  CTGGCTCTACTCAGGCTCATTG  ATCGAGGAAGCGGGTGGTGGT  TGATTGCTGCTCGGTTACAGAA  GTTATACGCTTGCAGCCTTCAG  TTCCAAAATGGCAGGGCTTA  GCATGGATGGAGCTCCTTATCT  GATCTGCAGTGGAAGAGACAAG  ATCTTCACACCGACGGTAAGTC  TCTTAACGTTTCCGATGGAGCC  TGTGATGACTCGGCTGATATTGA  GTTCAACATTGTCTCTCCTGG  GATCTTCTACAGCCTGAGAGAAC  CTCGATGGCCTCAACCTTTA  GGAGAGCAGTGACACCATCGA | ACATGGCAGGGACATTGAAAG  TGTCTTCCCAGTAACCATCATATAGG  TCGTAGAACACACAGGGAATGC  CCAGGCGGTTTTCCCTGCGAG  CACATTCATCAGCTTGAAGCAG  CGATAGGTCTAGTTCACTCTGC  TCGTACTCGGTCAAGGGAAGA  GCATCCAATCTCCATCTTTGTC  ATCATCTCCACCGATAACCACC  GTCAGATCTTCCACCCTGTTCA  CTCTTCCAGCGAAACTAAACCG  AGCAACAAGAGCCCAAATGAGA  GCTGTAGATGAGCTCCTCGAT  CTGTACACTCTCTGGTAACTCTG  ATGTTGCTCTCGGCTTCTGT  GCCAAGGTACGACCATCTTCA | 120  94  119  119  102  99  100  171  109  155  167  100  109  117  162  120 | 104.3  101.3  98.2  98.2  99.9  99.9  104.7  101.0  95.1  110.1  109.7  103.4  103.3  99.8  104.7  103.8 |

**Supplementary Table 2:** Comparisons between data sets obtained from flight experiments, clinorotated samples (both at KSC and UL) in the presence and absence (0 mag) of HGMFs. The axes assignments of each factor correspond with all figures shown in the manuscript and supplements.

| Axis: | Y | | | | | | | | |
| --- | --- | --- | --- | --- | --- | --- | --- | --- | --- |
| X |  | Flight-HGMF | Flight 0 mag | Clino HGMF | Clino  0 mag | static HGMF | static 0 mag | UL Clino HGMF | UL Clino 0mag |
|  | Flight-HGMF | - | #1 | #2 | #3 | #4 | #5 | #6 | #7 |
|  | Flight-0mag | #1 | - | #8 | #9 | #10 | #11 | #12 | #13 |
|  | Clino HGMF | #2 | #8 | - | #14 | #15 | #16 | #17 | #18 |
|  | Clino 0-mag | #3 | #9 | #14 | - | #19 | #20 | #21 | #22 |
|  | static HGMF | #4 | #10 | #15 | #19 | - | #23 | #24 | #25 |
|  | static 0mag | #5 | #11 | #16 | #20 | #23 | - | #26 | #27 |
|  | UL Clino HGMF | #6 | #12 | #17 | #21 | #24 | #26 | - | #28 |
|  | UL Clino 0 mag | #7 | #13 | #18 | #22 | #25 | #27 | #28 | - |

**
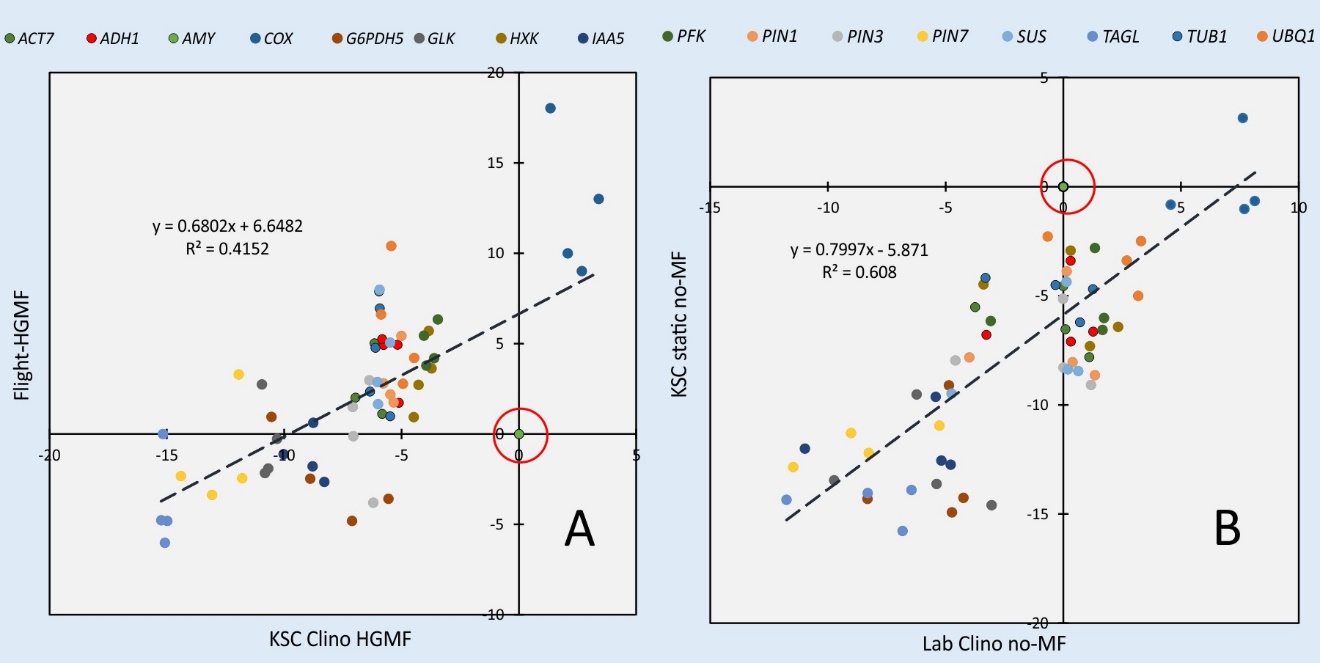
**

**Supplementary Figure 3:** Transcription values between space grown *Brassica rapa* seedlings and clinorotated ground controls (A) show strong reduction of amylase transcription in the presence and absence of magnetic fields. A comparison between static and clinorotated seedlings (B) shows higher *AMY1* levels in static samples. The data are identical to manuscript Fig. 5 but are based on *AMY1* as reference (circled). *AMY1* transcription was significantly different from the average of all examined measurements (n = 64); p-values for *AMY1* = 0.004 and <0.0001 for A and B, respectively.


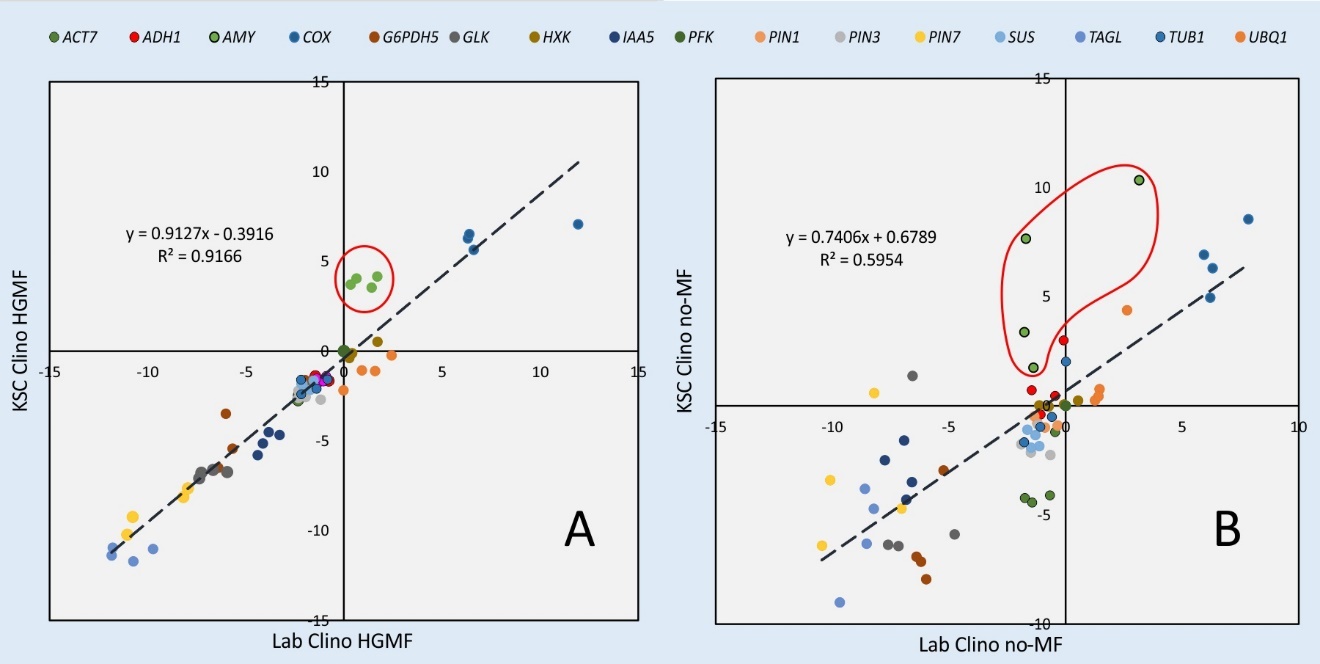


**Supplementary Figure 4:** Transcription values between *Brassica rapa* seedlings clinorotated in the flight hardware at the KSC vs. clinorotation in the lab show enhanced *AMY1* transcription in the data derived from the KSC samples in the presence (A, #17) and absence (B, #22) of magnetic fields. Reference is *PFK*; p-values for *AMY1* = 0.002 and 0.003 for the A and B, respectively.

**
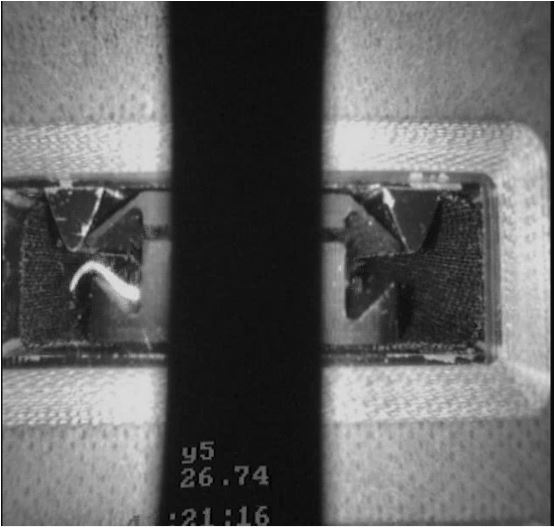
Supplementary Video 1**: The deflection of root growth by HGMF are documented in a time-laps video. The root curves away from the HGMF-inducing wedge.

**
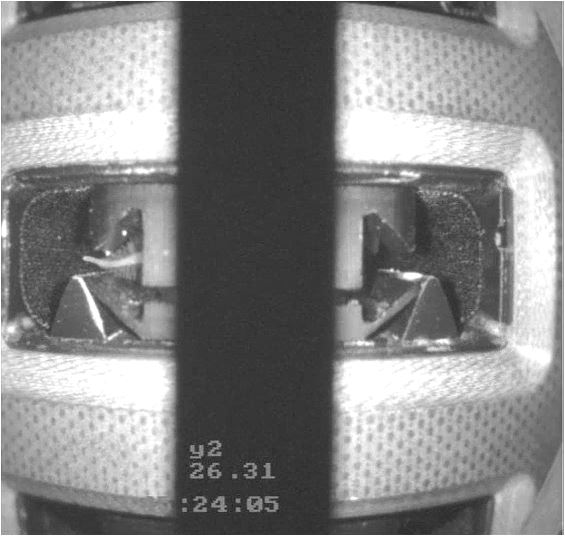
Supplementary Video 2**: Time lapse documentation of the deflection of root growth by HGMF during clinorotation. The jitter that resulted from flexing equipment has been eliminated during post-processing but is noticeable through the jitter of the video overlay.
